# Continuous Infusion Versus Bolus Injection of Loop Diuretics for Patients With Congestive Heart Failure: A Meta-Analysis

**DOI:** 10.7759/cureus.34758

**Published:** 2023-02-08

**Authors:** Jithin Karedath, Anim Asif, Niharika Tentu, Tafseer Zahra, Saima Batool, Meenakshi Sathish, Qudsia I Sandhu, Areeba Khan

**Affiliations:** 1 Internal Medicine, James Cook University Hospital, Middlesbrough, GBR; 2 Medicine, Fauji Foundation, Rawalpindi, PAK; 3 Internal Medicine, Liaquat National Hospital, Karachi, PAK; 4 Medicine, California Institute of Behavioral Neurosciences & Psychology, Fairfield, USA; 5 Internal Medicine, Hameed Latif Hospital, Lahore, PAK; 6 Surgery, Caribbean Medical University School of Medicine, Chicago, USA; 7 Medicine, Dera Ghazi Khan Medical College, Dera Ghazi Khan, PAK; 8 Critical Care Medicine, United Medical and Dental College, Karachi, PAK

**Keywords:** continuous infusion, acute decompensated heart failure, loop diuretic, bolus administration, furosemide

## Abstract

Loop diuretics continue to be a crucial component of pharmacological treatment, to eliminate extra fluid and enhance symptom control in acute decompensated heart failure (ADHF). Understanding the loop diuretics' more efficient form of administration would be very beneficial in improving the management of people's ADHF, resulting in a quicker resolution of symptoms and a notable decrease in morbidity. To assess the outcomes of intravenous continuous infusion with bolus injection of loop diuretics for patients with ADHF, this meta-analysis was carried out. The current meta-analysis was conducted as per the Cochrane Collaboration guidelines and the Preferred Reporting Items for Systematic Reviews and Meta-Analysis extension (PRISMA) guidelines. A search was carried out on PubMed and EMBASE databases for studies comparing continuous infusion with intermittent bolus injection of furosemide in patients with congestive heart failure without restriction on the language of publication from 1 January 2001 to 31 July 2022. The primary outcome of the meta-analysis was all-cause mortality and loss of body weight (kg). Pre-defined secondary outcomes included length of hospital stay (LOS) in days, brain natriuretic peptide (BNP) reduction (pg/ml), number of patients with hypokalemia, and urine output at 24 hours (ml). A total of nine articles were included in this meta-analysis enrolling 713 patients. No significant difference was reported between patients who received intermittent bolus injections and continuous infusion of furosemide in regards to all-cause mortality, LOS, total urine output, the incidence of hypokalemia, and change in BNP. However, the reduction of body weight was greater in the continuous infusion group compared to bolus administration. In conclusion, in the current meta-analysis of nine randomized controlled trials (RCTs), continuous infusion of furosemide seemed to have a greater reduction of body weight. However, no significant difference was there in 24-hrs urine output. However, we cannot conclude that intravenous continuous infusion has a better diuretic effect compared to bolus administration.

## Introduction and background

Heart failure (HF) is a complex clinical syndrome, caused by functional or structural cardiac abnormality, or both, leading to increased intra-cardiac pressures during exertion or at rest or decreased cardiac output or both [1]. In high-income nations, HF is also the primary reason for hospitalization for patients over 65 years [2]. Due to emergency room visits, readmissions, and extended hospital stays, this chronic illness represents a significant cost for healthcare systems worldwide [3].

Fluid retention is a classic effect of cardiac failure that occurs due to impaired or normal contraction of the heart [4]. The mainstay of treatment for fluid overload in HF is loop diuretics. They are recognized as the only drugs that, when administered intravenously (IV), may effectively decrease fluid retention in HF within a short period [1]. It is the preferred therapy for decongestion in patients with decompensated HF [5].

Despite the recommended use of IV loop diuretics, no consensus is there in regard to dosage and mode of administration in individuals with HF. This is at the physicians' discretion, which differs among medical facilities and nations [6]. These medications produce a strong and quick diuresis when administered IV as bolus injections, which is the conventional method of delivery. However, a number of issues with this administration strategy have been brought out [7]. It has been suggested that administering these diuretics in intermittent bursts could cause significant variations in intravascular volume and increased peak serum levels, enhancing their toxicity [8]. Additionally, it has been suggested that those with significant right ventricular dysfunction, cardiorenal syndrome, or diuretic resistance may benefit more from the continuous mode of administration [9]. When immediate decongestion is needed or when bolus injections failed to sufficiently decongest patients with HF, doctors frequently employ continuous infusion [10]. However, a lack of consensus and clarity is there on the preferences in international guidelines for cardiac failure. The European Society of Cardiology (ESC) guidelines for HF diagnosis and management released in 2021 include recommendations for the use of diuretics in the management of acute heart failure (AHF). These guidelines recommended that furosemide can be given as two to three daily boluses or as a continuous infusion. Daily single bolus administrations are discouraged because of the possibility of post-dosing sodium retention [11]. However, no consensus is found among international guidelines [5]. Therefore, it is important to analyze the currently available literature regarding the use of diuretics. Understanding the more effective mode of administration of loop diuretics would be very helpful in enhancing the management of individuals with acute decompensated heart failure (ADHF), leading to faster resolution of symptoms and a significant reduction in mortality and morbidity. Therefore, this meta-analysis was conducted to compare the effects of IV continuous infusion versus bolus injection of loop diuretics for patients with ADHF.

## Review

Methodology

The current meta-analysis was conducted as per the Cochrane Collaboration guidelines and the Preferred Reporting Items for Systematic reviews and Meta-Analysis extension (PRISMA) guidelines.

Search Strategy and Inclusion Criteria

A search was carried out on PubMed and EMBASE databases for studies comparing continuous infusion with intermittent bolus injection of furosemide in patients with congestive HF without restriction on the language of publication from 1 January 2001 to 31 July 2022. The search was conducted using the following key terms: “continuous infusion”, “bolus injection”, “loop diuretics”, and “acute decompensated heart failure”. MeSH (Medical Subject Headings) was also used. The above key terms were combined using Boolean operations. The reference list of all included articles was screened for a comprehensive search. The search was done by two authors independently and it was reviewed by the principal investigator.

Two independent reviewers screened all titles and abstracts of studies to identify potentially eligible articles. The full-text articles of eligible were evaluated to assess whether they are suitable for inclusion in the meta-analysis. Any discrepancy between the two reviewers was resolved through discussion or the involvement of the third author.

Eligibility Criteria

We included all randomized clinical trials (RCTs) comparing continuous infusion with intermittent bolus injection of furosemide in patients with congestive HF that were published in 2001 or onwards. We excluded studies that only recruited patients with chronic stable HF. Case reports, case series, observational studies, non-randomized studies, and reviews were excluded. Studies including valvular heart disease, post-cardiac surgical patients, and congenital heart disease were also excluded from the current meta-analysis.

Outcomes and Data Extraction

The primary outcome of the meta-analysis was all-cause mortality and loss of body weight (kg). Pre-defined secondary outcomes included length of hospital stay (LOS) (days), brain natriuretic peptide (BNP) reduction (pg/ml), number of patients with hypokalemia, and urine output at 24 hours (ml).

Two authors independently extracted data from all included studies. Data included the author's name, year of publication, dose of drugs, sample size, and outcome measures. Any discrepancy between the two reviewers was resolved through discussion or the involvement of the third author.

Risk of Bias Assessment

The risk of bias of each individual was assessed by two authors independently using the Cochrane Risk-of-Bias. This tool evaluates seven domains that included “random sequence generation, allocation concealment, masking of participants and personnel, blinding of outcome assessment, incomplete outcome data, selective outcome reporting, and other sources of bias”. Any disagreement between the two reviewers was resolved through discussion or the involvement of the third author.

Statistical Analysis

Data analysis was done using Review Manager Version 5.4.0 (The Cochrane Collaboration, Copenhagen, Denmark). For categorical outcomes, data were pooled using a random or fixed effect effects model, according to the Mantel-Haenszel model. The risk ratio (RR) with 95% confidence intervals was used as a measure of the treatment effect of all-cause mortality and hypokalemia. For continuous outcomes, the mean difference was determined along with their standard deviation. A p-value of less than or equal to 0.05 was considered statistical significance for each outcome of interest. Statistical heterogeneity of each outcome was determined using the I-square statistics that describes the percentage of total variation across the articles because of heterogeneity instead of chance. An I-square value of 25%, 50%, and 75% showed a low, moderate, and high degree of heterogeneity, respectively. In the case of an I-square value of more than 50%, a random effect model was used, while a fixed effect model was used if the I-square value was less than 50%.

Results

**Figure 1:**
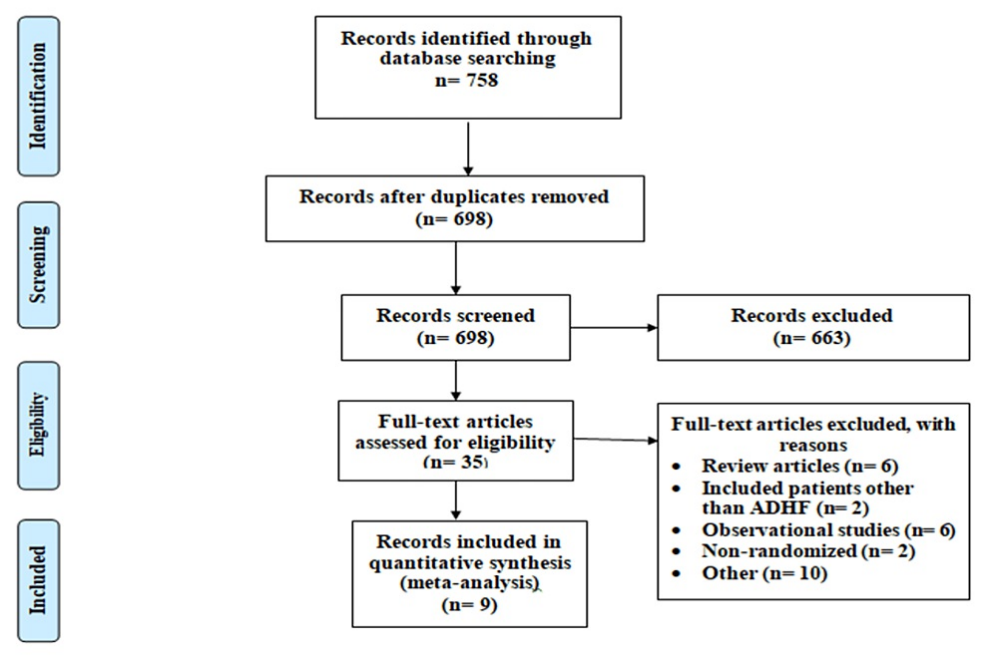
PRISMA flowchart of selection of studies ADHF: acute decompensated heart failure

Figure 1 shows the PRISMA flowchart of selection studies. Out of a total of 758 studies results from the initial database search, 698 articles were retrieved for abstract and title screening. Full texts of 35 articles were accessed to assess the eligibility criteria. In the end, nine articles fulfilled the inclusion criteria enrolling 786 patients included in this meta-analysis.

**Figure 2:**
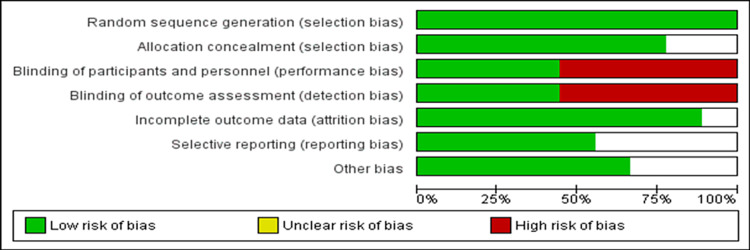
Risk of bias assessment

Table 1 shows the characteristics of studies included in the current meta-analysis [6,10,12-18]. Among all RCTs included in this meta-analysis, three were multicenter [6,10,16]. The pooled mean age of patients was 66.86 years. Nearly three-quarters of patients were male. Figure 2 shows the risk of bias assessment of all included studies. Three studies were double-blinded [6,13,16], and two studies were single-blinded [10-14].

**Table 1:** Characteristics of the included studies HTN: hypertension

Author	Year	Setting	Study groups	Dose	Sample size	Mean age	Males (n)	Diabetes (n)	HTN (n)
Allen et al. [12]	2010	Single center	Continuous infusion	162 (52) mg per day	20	59.5	26	24	32
			Bolus injection	162 (48) mg per day	21				
Felker et al. [6]	2011	Multicenter	Continuous infusion	80 (40-140) mg per day	152	66	226	158	NR
			Bolus injection	80 (60-160) mg per day	156				
Llorens et al. [10]	2014	Multicenter	Continuous infusion	280 mg per day	36	82	32	57	86
			Bolus injection	120 mg per day	37				
Palazzuoli et al. [13]	2014	Single center	Continuous infusion	80 mg per day	43	79.5	40	48	73
			Bolus injection	80 mg per day	39				
Shah et al. [14]	2014	Single center	Continuous infusion	100 mg per day	30	58.22	66	49	53
			Bolus injection	100 mg per day	30				
Shree et al. [15]	2021	Single center	Continuous infusion	24-36 mg per day	28	66	32	12	14
			Bolus injection	120 mg per day	28				
Thomson et al. [16]	2010	Multicenter	Continuous infusion	197 (148) mg per day	26	55.5	32	NR	NR
			Bolus injection	172 (97) mg per day	30				
Yayla et al. [17]	2015	Single center	Continuous infusion	240 mg per day	15	68.55	15	12	22
			Bolus injection	160 mg per day	14				
Zheng et al. [18]	2021	Single center	Continuous infusion	160 mg per day	42	66.47	53	38	76
			Bolus injection	200 mg per day	39				

**Figure 3:**
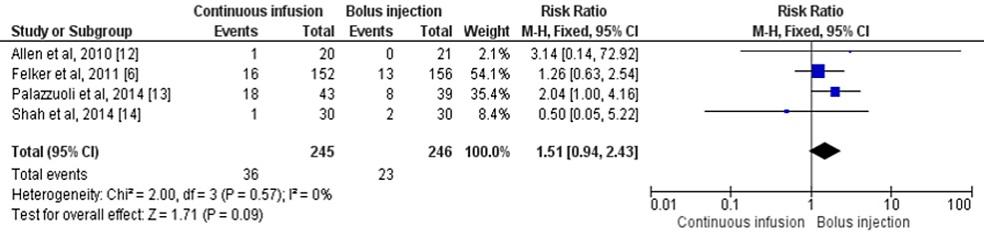
Forest plot of all-cause mortality Sources: References [6,12-14] CI: confidence interval

Four studies compared all-cause mortality among patients who received intermittent bolus injections and continuous infusion of furosemide within six months [6,12-14]. The incidence of all-cause mortality was higher in patients receiving continuous infusion (14.69%) compared to patients receiving bolus injection (9.34%), but no significant differences were reported between the two groups (RR: 1.51, 95% CI: 0.94-2.43, I-square= 0%) as shown in Figure 3. Low heterogeneity was there in pooled effect.

**Figure 4:**
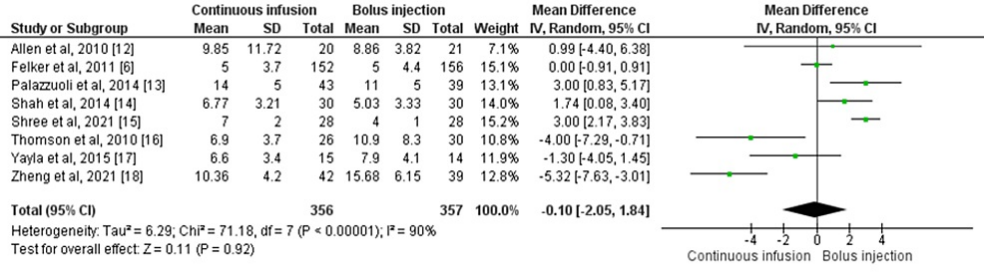
Forest plot of length of hospitalization Sources: References [6,12-18] SD: standard deviation; IV: intravenous; CI: confidence interval

Eight studies assessed the LOS by enrolling a total of 713 patients [6,12-18]. No significant difference was found in the LOS between the continuous infusion and bolus injection groups (mean difference: -0.10, 95% CI: -2.05-1.84, I-square: 90%) as shown in Figure 4. High heterogeneity was found across studies.

**Figure 5:**
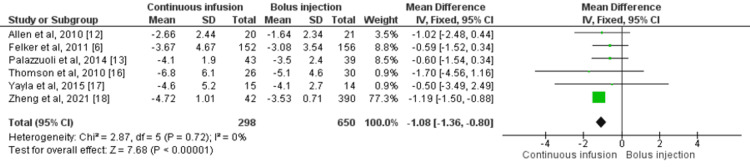
Forest plot of reduction in body weight (kg) Sources: References [6,12,13,16-19] SD: standard deviation; IV: intravenous; CI: confidence interval

Six RCTs compared reduction in body weight as an outcome of diuretic effect between patients who received intermittent bolus injection and continuous infusion of furosemide [6,12,13,16-19]. Continuous infusion of furosemide resulted in a greater reduction of body weight as compared to bolus administration of furosemide (mean difference: -1.08, 95% CI: -1.36, -0.80, I-square: 0%) as shown in Figure 5. Low heterogeneity was found in pooled effect.

**Figure 6:**
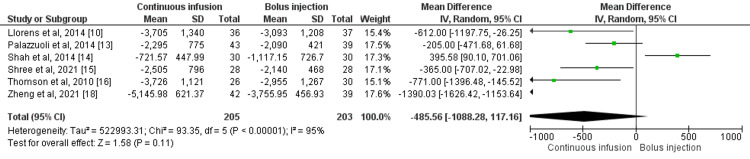
Forest plot of total urine output in 24 hours (ml) Sources: References [10,13-16,18] SD: standard deviation; IV: intravenous; CI: confidence interval

Eight studies compared total urine output in the 24 hours between patients who received intermittent bolus injections and continuous infusion of furosemide [10,13-16,18]. No significant difference was found in regards to total urine output between the two study groups (mean difference: -485.56, 95% CI: -1088.28, 117.16I-square: 95%) as shown in Figure 6. Statistical heterogeneity was assessed as high among the study results.

**Figure 7:**
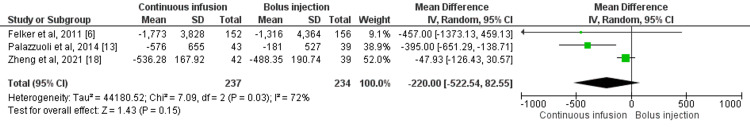
Forest plot for reduction of BNP Sources: References [6,13,18] SD: standard deviation; IV: intravenous; CI: confidence interval; BNP: brain natriuretic peptide

Three studies compared the reduction in BNP between the study groups [6,13,18]. No significant difference was found in regard to change in BNP between the two study groups (mean difference: -220, 95% CI: -522.54, 82.55 I-square: 72%) as shown in Figure 7. Statistical heterogeneity was assessed as moderate among the study results.

**Figure 8:**
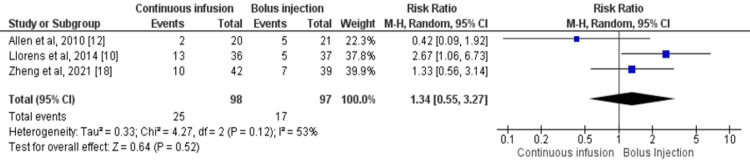
Forest plot of hypokalemia Sources: References [10,12,18] CI: confidence interval

Three studies assessed the incidence of hypokalemia on day 3 or at discharge in patients who received intermittent bolus injections and continuous infusion of furosemide [10,12,18]. The incidence of hypokalemia was lower in patients receiving intermittent bolus injection (17.52%) compared to the continuous infusion (25.51%) but no significant difference was reported between the two groups (RR: 1.35, 95% CI: 0.55-3.27, I-square: 53%) as shown in Figure 8. Statistical heterogeneity was assessed as moderate among the study results.

Discussion

The current meta-analysis aimed to compare the effects of IV continuous infusion versus bolus injection of loop diuretics for patients with ADHF. Our findings showed that no significant difference was found regarding the LOS and all-cause mortality. Continuous infusion of furosemide was associated with greater weight loss compared to patients receiving bolus injections. We did not identify any significant difference in plasma creatinine at day 3 or discharge, change in BNP, the incidence of hypokalemia, and total urine output in 24 hours between the two groups. A meta-analysis conducted by Ng et al. did not report any significant difference in plasma creatinine at day 3 or discharge, the incidence of hypokalemia, LOS, and all-cause mortality [19]. However, this trial reported a significant reduction in BNP levels in patients receiving continuous infusions of furosemide [19]. Our meta-analysis included two additional studies conducted in 2021 [15,18].

To affect diuresis, all loop diuretics have to be secreted and filtered to their site of action on the luminal side of the loop of Henle [20]. Theoretically, a loop diuretic administered continuously might keep levels over the natriuretic threshold and give more consistent diuresis without significant changes in intravascular volume. These qualities can eliminate two of the recognized mechanisms of diuretic resistance [21]. Despite the theoretical benefits mentioned above, we did not find any significant benefits associated with the utilization of continuous infusion of furosemide in patients with congestive HF.

Several studies have shown that continuous infusion was associated with lesser adverse effects and short hospital stay compared to bolus injection [7,22]. Considering the high heterogeneity of our meta-analysis in some of the outcomes, certain confounding factors like concurrent medications, small sample, age, and comorbidities may have affected the duration of hospital stay in all included RCTs that assessed duration of hospital stay. We were unable to adjust for these factors at the review level. Thus, the findings of this meta-analysis should be interpreted cautiously.

Reduction of body weight after administration of furosemide is linked with enhanced outcomes [23]. The current meta-analysis found that patients receiving continuous infusions of furosemide were associated with a greater reduction of body weight compared to patients receiving bolus injections. As not all patients were catheterized to assure proper measurement and recording, body weight may be a better substitute for total urine production as a measure of the diuretic impact [19]. We believe that body weight may be a better surrogate measure for diuretic effect than total urine output, as not all patients were catheterized to ensure accurate measurement and recording. There was potential reporting bias, as observed in an RCT by Shah et al. in 2014 [14]. While weight assessment might appear simple, it is technically challenging in practice and not feasible in many patients with HF. Additionally, there is a poor correlation between weight loss and fluid output [24].

In reaction to volume expansion and potential increased wall stress, the ventricular wall of the heart releases a hormone called BNP [25]. Decreases in BNP levels, particularly more than 30% from levels at admission are associated with long terms as well as short-term clinical outcomes [26]. Consistent plasma diuretic concentrations from continuous infusion are thought to reduce neuro-humoral activation, sodium reabsorption and rebound water, and vasoconstriction of efferent renal arterioles, all of which contribute to a superior diuretic effect. It might also lead to a higher reduction in BNP [27].

In the current meta-analysis, we did not find any significant difference between intermittent bolus and continuous infusion in terms of reduction in BNP levels, in contrast to a meta-analysis conducted by Ng et al. [19] who found a greater reduction of BNP in patients receiving continuous infusion compared to patients receiving bolus injection. Our meta-analysis included a study conducted by Zheng et al. [18]. Out of the three studies included, only Palazzuoli et al. [13] showed greater BNP reduction in patients receiving continuous infusion. Possible confounding factors to the changes in BNP could be distinct diuretic doses given in these three RCTs [12,13,18]. A study conducted by Frea et al. also did not have any effect on the mode of administration of BNP reduction [28]. However, further studies are required including a larger sample size to warrant these findings.

The current meta-analysis has important limitations. Firstly, all studies included in this meta-analysis were having differences in characteristics of patients, a small sample size with only one study out of the nine studies in this meta-analysis having more than 100 patients included, and different doses of diuretics and schedules of administration. In this review, we were unable to control for confounding factors. All these factors might have increased the heterogeneity of certain outcome variables. In the future, studies need to be conducted with a larger sample size to enhance the power of the results.

## Conclusions

In conclusion, in the current meta-analysis of nine RCTs, continuous infusion of furosemide seemed to have a better greater reduction in body weight. However, no significant difference was there in 24-hrs urine output, LOS, all-cause mortality, the incidence of hypokalemia, and BNP reduction. We cannot conclude that IV continuous infusion has a better diuretic effect compared to bolus administration. However, considering the small sample size of included studies, the findings need to be interpreted with caution. In the future, more RCTs need to be conducted by enrolling a large number of patients to enhance the power of the results that will make them more generalizable.
